# A Probability Co-Kriging Model to Account for Reporting Bias and Recognize Areas at High Risk for Zebra Mussels and Eurasian Watermilfoil Invasions in Minnesota

**DOI:** 10.3389/fvets.2017.00231

**Published:** 2018-01-04

**Authors:** Kaushi S. T. Kanankege, Moh A. Alkhamis, Nicholas B. D. Phelps, Andres M. Perez

**Affiliations:** ^1^Department of Population Medicine, College of Veterinary Medicine, University of Minnesota, Minneapolis, MN, United States; ^2^Faculty of Public Health, Health Sciences Center, Kuwait University, Kuwait City, Kuwait; ^3^Environmental and Life Sciences Research Center, Kuwait Institute for Scientific Research, Kuwait City, Kuwait; ^4^Department of Fisheries, Wildlife and Conservation Biology, College of Food, Agriculture and Natural Resource Sciences, University of Minnesota, Minneapolis, MN, United States; ^5^Minnesota Aquatic Invasive Species Research Center, University of Minnesota, Minneapolis, MN, United States

**Keywords:** risk assessment, spatial modeling, geostatistics, early detection, surveillance, reporting, observation bias

## Abstract

Zebra mussels (ZMs) (*Dreissena polymorpha*) and Eurasian watermilfoil (EWM) (*Myriophyllum spicatum*) are aggressive aquatic invasive species posing a conservation burden on Minnesota. Recognizing areas at high risk for invasion is a prerequisite for the implementation of risk-based prevention and mitigation management strategies. The early detection of invasion has been challenging, due in part to the imperfect observation process of invasions including the absence of a surveillance program, reliance on public reporting, and limited resource availability, which results in reporting bias. To predict the areas at high risk for invasions, while accounting for underreporting, we combined network analysis and probability co-kriging to estimate the risk of ZM and EWM invasions. We used network analysis to generate a waterbody-specific variable representing boater traffic, a known high risk activity for human-mediated transportation of invasive species. In addition, co-kriging was used to estimate the probability of species introduction, using waterbody-specific variables. A co-kriging model containing distance to the nearest ZM infested location, boater traffic, and road access was used to recognize the areas at high risk for ZM invasions (AUC = 0.78). The EWM co-kriging model included distance to the nearest EWM infested location, boater traffic, and connectivity to infested waterbodies (AUC = 0.76). Results suggested that, by 2015, nearly 20% of the waterbodies in Minnesota were at high risk of ZM (12.45%) or EWM (12.43%) invasions, whereas only 125/18,411 (0.67%) and 304/18,411 (1.65%) are currently infested, respectively. Prediction methods presented here can support decisions related to solving the problems of imperfect detection, which subsequently improve the early detection of biological invasions.

## Introduction

Aquatic invasive species (AIS) have the potential to affect animal, environmental, and public health (1, 2). The state of Minnesota in the United States has experienced numerous AIS incursions and spend over 10 million dollars each year on activities intended to prevent, control, or manage AIS (3, 4).

Zebra mussels (ZMs) (*Dreissena polymorpha*) and Eurasian watermilfoil (EWM) (*Myriophyllum spicatum*) are AIS of concern for Minnesota and have been reported in Minnesota since 1989 and 1987, respectively (5). The first introduction of ZMs into North America is attributable to ballast water from transatlantic ships (6). ZMs are rapidly propagating bivalves that disrupt the stability of the food web in aquatic ecosystems affecting both pelagic and benthic species (7). Removal of ZMs colonizing public water supply pipes and pipes of industrial facilities has cost nearly $267 million in the ZM affected region in North America between 1989 to 2004 period (8). Similarly, EWM, an invasive aquatic macrophyte, was likely introduced into North America through aquarium trade (6). EWM proliferates rapidly impeding the effective removal or control strategies upon establishment in a waterbody (9). Dense vegetation of EWM outcompetes native macrophytes and interrupts recreational activities (9). An intensive hand harvesting project to control EWM, conducted in the upper Saranac Lake in New York, reported a labor cost of $351,748/year in that one lake alone (10).

Aggressive and costly programs have been implemented in Minnesota to control AIS (3). For example, since 2014, $10 million per year has been allocated by the Minnesota legislature to provide resources for county-based AIS prevention activities, such as education, surveys, and watercraft inspections (4). However, because the risk of AIS invasion had not been previously quantified, the resources were distributed proportionally to the share of boat ramps and trailer parking spaces in each county (4). The funds are invested on prevention of the introduction or limitation of the spread of AIS within the county (3, 4). Because of the high economic and conservation burden posed by the invasions, forecasting of the areas at high risk for invasions is an urgent research priority (2).

The two AIS have been invading Minnesota waters for approximately 30 years; therefore, the measurement of propagule pressure, i.e., the “introduction effort,” needs to be focused at the local scale such as at individual waterbody (11). As a solution, previous studies have suggested using surrogate variables such as the number of boat ramps and distance to the major roads in the absence of waterbody-specific data when measuring the propagule pressure (12). One of the most challenging waterbody-specific variables is the measurement of human-mediated dispersal (9, 12, 13). Use of human population density as a proxy for the human-mediated dispersal may serve as a solution. However, densely populated areas may also tend to report the invasions more frequently, compared to less populated areas (14),^1^ which may also lead to reporting bias and underreporting.

The objective of this study was to estimate the potential range expansion of ZMs and EWM in Minnesota, using a combination of network analysis and co-kriging, a spatial interpolation technique to account for underreporting. The advantage of using co-kriging is that the technique enables the prediction of values for the locations without observed data, using other correlated and highly sampled variables (15, 16). Co-kriging is commonly used in gold mining and lake and reservoir studies, and has rarely been used in veterinary epidemiological and public health studies as well (17–20). Environmental conservation studies, such as the controlling the spread of invasions, often suffer from lack of data and reporting bias because of the financial constraints on surveillance (1). In Minnesota, invasions are often reported by volunteers and the presence of the AIS may be missed in some waterbodies due to insufficient coverage, which decreases the sensitivity of the reporting. The specificity of the reporting system, instead, may be considered acceptable, given that false positive cases are unexpected. False positives are unlikely because, the Minnesota Department of Natural Resources (MNDNR) confirms newly reported invasions prior to adding them to the official online database of infested waters (5). Consequently, the limitation of this passive surveillance system is the potential underreporting of the conditions. Co-kriging may also compensate for the reporting bias and underreporting by augmenting the predictive power of one variable with the support of other correlated and highly sampled variables.

Recognition of areas at high risk may act as an early warning system and help the prioritization of waterbodies for a targeted and efficient allocation of limited resources to improve both defensive and offensive management strategies (21, 22). Such risk targeted approaches certainly represent improvements over the random selection of waterbodies for surveillance and management purposes (23, 24). For example, current guidelines for conducting AIS early detection and baseline monitoring in lakes of Minnesota suggest that volunteers select waterbodies based on factors such as public water access, boater traffic, tourist activity, etc. (25). However, selecting waterbodies based on multiple criteria is challenging and we propose that a method which take all the most relevant risk factors into account and provide a risk rank would be a better fit to guide the volunteers. Study results may inform risk-based surveillance and management of invasions (21, 23), a process defined as making decisions for identifying, evaluating, selecting, prioritizing, and implementing control measures (26). This work demonstrates the use of analytical models to estimate risk while accounting for reporting bias, with the ultimate objective of evaluating and modifying the policies and practices on biological invasions (23).

## Materials and Methods

### Study Area and AIS Presence Data

A total of 18,411 point locations representing waterbodies of Minnesota were considered as the study population in this study. Waterbodies were mainly lakes and ponds (*n* = 18,263) and were represented by the centroids of each waterbody. In addition to the lakes, several riverine locations (*n* = 148) from major rivers were included in the analysis. Riverine locations were identified at the rivers’ midpoint within each county. The locational data for the waterbodies were extracted from the GIS layer referred to as “MNDNR Hydrography,” which is available from the Minnesota GIS Commons (27). Presence data for confirmed AIS locations were collected from the MNDNR database (5). By the end of 2015, there were 125/18,411 (0.67%) ZMs and 304/18,411 (1.65%) EWM infested waterbodies in Minnesota (5, see text footnote 1). The confirmed presence of the AIS was used in the study regardless of the magnitude of infestation, because assessments on the magnitude of infestation are not available.

### Waterbody-Specific Variables

Waterbody-specific variables (*n* = 6), were used as predictors in the co-kriging models. The six waterbody-specific variables included (1) ZMs or (2) EWM invaded waterbody, (3) connectivity to another ZM and (4) EWM invaded waterbody *via* a stream or a river, (5) boater traffic between waterbodies, and (6) inverse of the Euclidean distance to the nearest major road. Status of the invasions, i.e., confirmed presence of invasion was the primary variable for each AIS (variables 1 and 2). For the validation purposes, models were fit for years 2010 and 2015; therefore, two sets of each variable were calculated. The number of waterbodies from which each variable is available varied over the time (Table 1). However, the same boater traffic variable was used in both 2010 and 2015 model fits because boater traffic was calculated based on a survey conducted in 2013, as described below. The Euclidean distance to the nearest major road variable was the same for both 2010 and 2015 assuming the major roads remained unchanged.

**Table 1:** Number of waterbodies with the characteristic of each variable by 2010 and 2015.

		Number of waterbodies by 2010	Number of waterbodies by 2015
1	ZM invasion status^a^	57	125
2	EWM invasion status^a^	251	304
3	Connectivity to another ZM invaded waterbody *via* a river or a stream^b^	2,392	3,658
4	Connectivity to another EWM invaded waterbody *via* a river or a stream^b^	3,129	3,715
5	Eigenvector centrality of the boater traffic network	1,376	1,376
6	Inverse of the Euclidean distance to the nearest major road	18,411	18,411

*^a^Presence only*.

*^b^Connected waterbodies only*.

Proximity and connectivity to infested waterbodies have been recognized as key risk factors leading to ZM and EWM invasions (9, 28). Because of the pairwise distance calculation for the semi-variance of candidate variables in the model, the kriging process includes the distance between locations as an integral part of the algorithm (15). Therefore, when AIS presence/absence is the primary variable, the spatial dependence, i.e., the distance to the nearest infested location is inherently included in the co-kriging model.

Surface water connectivity between waterbodies *via* a stream or a river was obtained by intersecting the map of the river and streamlines features with the polygon features representing lakes, ponds, and reservoirs using ArcGIS version 10.3.1 (29). River and streamline feature data were obtained from the “Stream Routes with Kittle Numbers and Mile Measures” GIS layer available from the Minnesota GIS Commons (30). Several published studies identified the potential for downstream (e.g., *via* downstream drift) and upstream (e.g., *via* watercraft) spread of ZMs and EWM (28, 31, 32). However, the distance measures denoting the extent of the spread upstream or downstream were either not studied or varied among the published literature. Therefore, for simplicity, an invasion was assumed to occur both up and down stream regardless of the flow direction. Invaded locations that were not directly intersecting a river or streamline were given a buffer distance of 100 m around the point location, and the closest river or stream feature was assigned as connected because the proximity to the infested location poses the risk of invasion (7, 9). Rivers and streams were represented by a unique identification number referred to as “Kittle Numbers” assigned by the MNDNR (30, 33). Kittle numbers consisted of an alphabetical letter, followed by a string of digits (33). For example, if an invaded waterbody was connected to kittle number #H026, then any waterbody connected to #H026 was assigned as connected to an invaded waterbody. Connectivity networks were generated separately for ZMs and EWM.

Boater traffic between waterbodies may lead to human-mediated dispersal of AIS (9, 13). Here, boater traffic was measured using data collected by the MNDNR Watercraft Inspection Program, a survey conducted since 1992 as a conservation measure to protect state waters (34). The Watercraft Inspection Program survey is conducted at selected waterbodies. Priority for data collection is given to those that are invaded, located near an invaded waterbody, highly used, or located close to popular travel destinations (34). The boaters who visit the waterbodies were interviewed regarding the previous waterbody visited and the waterbody they plan to visit next. In 2013, the Watercraft Inspection Program surveys were conducted at 240 locations, and 119 (49.6%) of those locations were invaded by either ZMs or EWM. Because of the miscellaneous reporting errors, only 21% of the surveys were eligible to be used in the final Watercraft Movement Network. Based on the survey, boater traffic data were available from 1,376 unique waterbodies (7.5% of the total waterbodies). Because the analysis was focused on predicting the current risk of invasions rather than understanding the impact of boater traffic on past invasions, it was assumed that movements recorded in 2013 were representative of movement patterns observed between 1987 and 2015.

Network analysis, which provides a framework to identify units that are frequently or intensely connected within the network and identify contact patterns (35), was applied to the Watercraft Inspection Program data from 2013. A total of 187,074 surveys were conducted between April 25, 2013 and November 30, 2013. Recreational boater movement data are not collected during the winter season (34). In the analysis, network “nodes” were the waterbodies and visits between waterbodies served as “edges.” Each completed survey accounted for two edges, representing the following links: (1) between the previously visited location and the surveyed location, and (2) between surveyed location and the next stated location that the watercraft would visit. Three centrality measures, namely, the Eigenvector, Betweenness, and Degree were calculated for the network. The centrality measure that highly correlates with the status of the invasions by ZM and EWM was chosen, upon calculating the Pearson correlation analysis. Eigenvector centrality was chosen as the network parameter representing the connectivity of each waterbody within the watercraft movement network. Eigenvector centrality is a representation of the relative importance of a node regarding its position and connectivity to other highly connected nodes in the network (35). It was assumed that highly connected nodes could play a major role in distributing AIS.

Distance to the nearest major road represents the convenience of accessibility to a waterbody. Boater traffic data are collected from limited waterbodies; however, an indirect measure of the potential visitations is the calculation of road accessibility (12, 36). Therefore, distance to the nearest major road from the waterbodies was calculated using the major roads map of 2012, available through the Minnesota Geospatial Commons and originated from the Department of Transportation (37). As defined in the metadata of the spatial layer, road classes including interstate highways, freeways, arterials, and major collectors were considered as major roads in the analysis (37). The inverse of the Euclidean distance was used as the variable when fitting the models.

### Data Analysis: Co-Kriging to Estimate the Probability of Introduction

Probability co-kriging was used to estimate the probability of ZM or EWM introduction into the waterbodies, conditional to the distance between locations and other waterbody-specific variables. Co-kriging is a linear weighted averaging method in which weights are selected to minimize the variance of the estimation error by accounting for the spatial correlation between the waterbody-specific variables; weights are dependent on the distance between sampled locations (15). In this study, multiple correlated waterbody-specific variables were used to estimate the spatial distribution of the dependent variable in the non-sampled locations (15). The primary variable subjected to co-kriging is the invasion status of ZMs or EWM. Therefore, the “sampled locations” were those confirmed to be infested, whereas “not sampled locations” were those that without infestation reports. The cross correlation between variables is used to improve the predictions because the predictions are derived from both primary and secondary variables (15). A complete description of the application of co-kriging is available elsewhere (15, 19).

Pearson correlation coefficient was calculated to determine the correlation between the six waterbody-specific variables. Variables with a correlation coefficient ≥0.1 were selected to be included in the co-kriging models. Multiple co-kriging models were fit for both ZMs and EWM separately. Each model included the primary variable, i.e., the status of the invasion and two correlated variables. All possible two-way combinations were fit. Considering the potential mutualism between ZM and EWM suggested by multiple studies (38, 39), the variable pairing also included the use of invasion status of ZMs as a correlated variable used in co-kriging model to predict Eurasian milfoil and *vice versa*. Model performance was evaluated using the area under the receiver operating characteristic curve (AUC), a plot of model sensitivity (true positives) and 1 − specificity (i.e., false positives) (40). AUC values lower than 0.7 are considered relatively inaccurate because the proportion of false and true positive results is not substantially different, whereas AUC values greater than 0.7 are generally considered appropriate (40). Models with AUC value greater than 0.7 were considered accurate in this study.

The variables contributing to the co-kriging model with highest AUC were chosen. Hence, final models consisted of the primary variable representing the invasion status of each AIS and two other waterbody-specific variables. AUC values were calculated for each of the co-kriging models by true validation, which was done by fitting models to the invasions by 2010 and validating using the invasions reported between 2011 and 2015. Results of the co-kriging analysis were the probability of finding an AIS invaded waterbody conditional to the presence of an invaded location in the proximity and the waterbody-specific variables incorporated into the model. Small lag sizes (e.g., 0.04 km) and few lags (e.g., *n* = 12) were used in the computation of the co-kriging semivariogram. The use of small lag size and few lags was intended to reduce the exponential increase in the influence of an infested location to the nearby cells, i.e., to reduce the effect of high spatial autocorrelation (15). The choice of the parameter values for the co-kriging attributes such as the anisotropy factor and the angle were based on the spatial cluster analysis and directionality tests for the data (see text footnote 1). The parameter values are summarized in Table S1 in Supplementary Material.

The performance of the final co-kriging models for ZMs and EWM was estimated based on the predictive powers of the candidate models. The predictive powers were measured estimating the sensitivity and specificity, and the AUC of the candidate models. In the context here, sensitivity and specificity reflect the ability of the model to predict invaded and not invaded waterbodies, respectively. Because the goal of the model was to predict potential infestations, high sensitivity, rather than high specificity, was targeted when optimizing the models. In addition to the true validation, the co-kriging models were cross validated using *k* fold cross validation (*k* = 5). Cross validation is a process in which a set of AIS infested locations were left out from the model fitting, and the fitted model output was used to estimate the probability of finding an AIS invasion at those left out locations (41). Eighty percent of the cases were used for the model training, and testing was done using the 20% of the withheld cases for each validation. To maintain the consistency, the co-kriging parameters recognized during the true validation were used when fitting the models for the cross validation.

### Interpretation of the Co-Kriging Outputs

**Figure 1:**
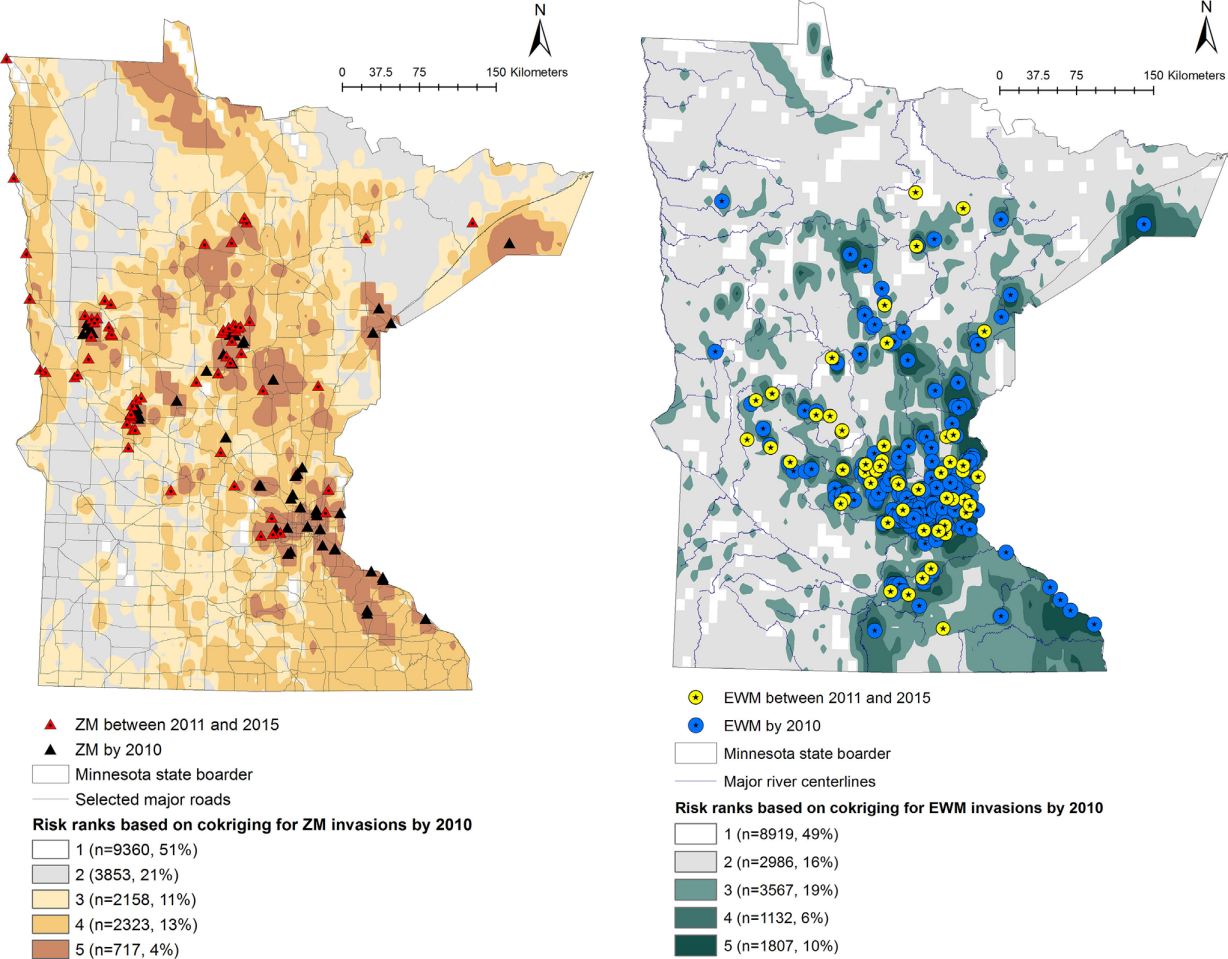
Co-kriging model outputs illustrating the probability of introduction of zebra mussels (ZMs) and Eurasian watermilfoil (EWM) to Minnesota waterbodies, for the invasions as of 2010. The risk classes 1 through 5 indicate the intensity of the probability of introduction, where class 5 represents a high probability of ZM or EWM introduction. The number of waterbodies under each category and as a percentage of the total waterbodies (*n* = 18,411) is listed.

**Figure 2:**
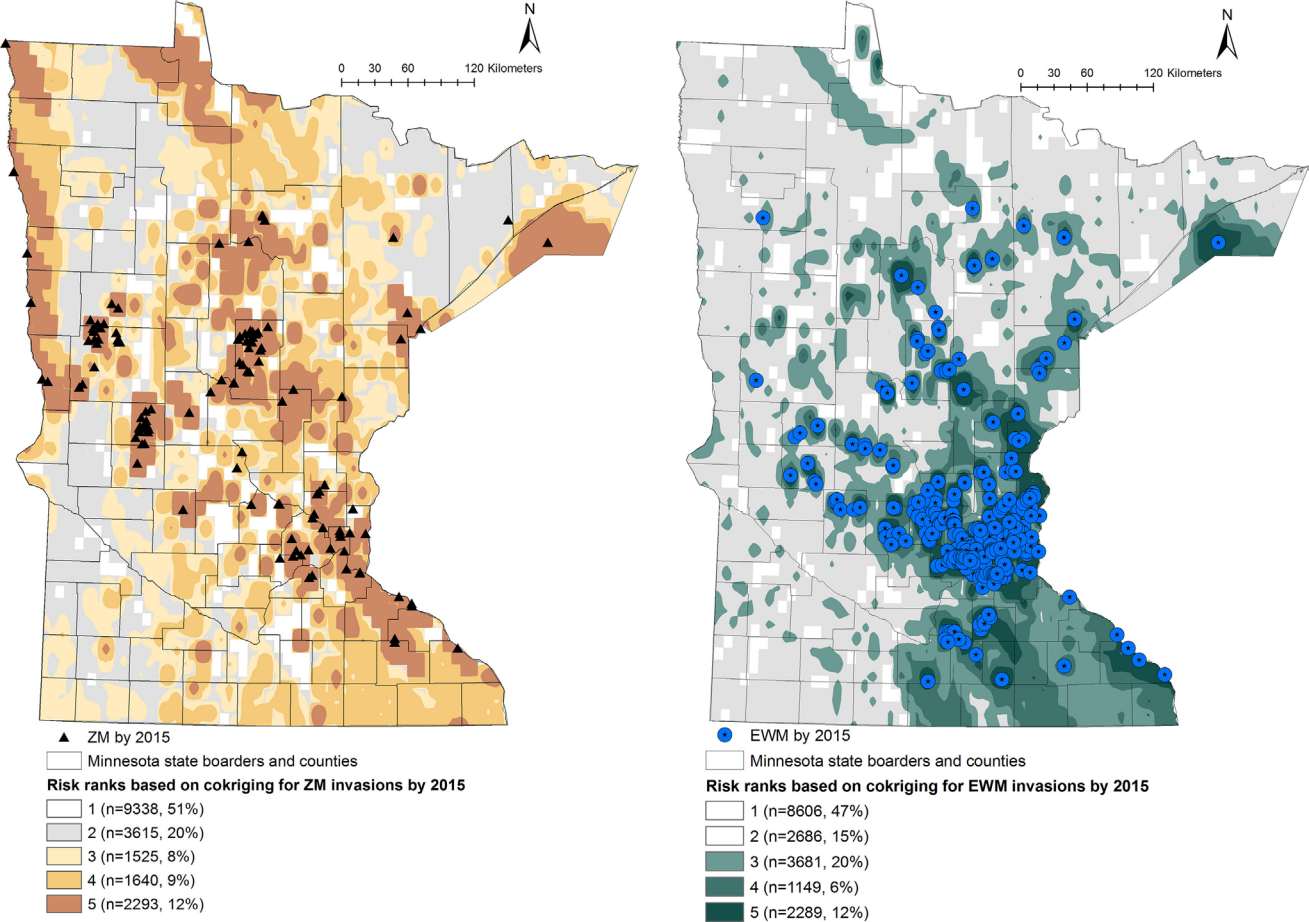
Co-kriging model outputs illustrating the probability of introduction of zebra mussels (ZMs) and Eurasian watermilfoil (EWM) to Minnesota waterbodies, for the invasion status of 2015. The risk classes 1 through 5 indicate the intensity of the probability of introduction, where class 5 represents a high probability of ZM or EWM introduction. The number of waterbodies under each category and as a percentage of the total waterbodies (*n* = 18,411) is listed.

Predicted probabilities were extracted for each of the waterbodies from the probability output of the co-kriging models, for ZMs and EWM separately. The outputs were ranked into five “risk rank” categories based on the quantiles of the output probability values. The risk ranks 1 through 5 were defined as follows: (5) very high, (4) high, (3) intermediate, (2) low, and (1) negligible risk of AIS introduction. The co-kriging risk rank resulting with highest sensitivity and specificity was considered the threshold for each model. The calculated probabilities of AIS invasion using co-kriging represent current risk status. In the absence of effective eradication measures to remove AIS from invaded waterbodies, the waterbodies that are currently recognized to be at risk will remain in the same status while the intensity of the risk of invasion may increase when newly AIS invasions are reported (Figures 1 and 2).

## Results

The Pearson correlation coefficients for each variable pair are summarized in Table 2. The variable pair with the highest AUC value for the true validation of the ZM model was the Eigenvector centrality of the watercraft movement network and the distance to the nearest major road (AUC = 0.78), whereas EWM was best predicted by the Eigenvector centrality of the watercraft movement network and the surface water connectivity to infested waterbodies (AUC = 0.76). The AUC values, sensitivity, and specificity at the threshold risk rank = 3 for the cross validations and true validation of co-kriging models are summarized in Table 3. The final model included the variables that were correlated with the invasion status and highly sampled.

**Table 2:** Pearson correlation coefficient for the six waterbody-specific variables used in the study.

		ZM invasion status (primary variable)	EWM invasion status (primary variable)
1	ZM invasion status	1.00	0.10
2	EWM invasion status	0.10	1.00
3	Connectivity to another ZM invaded waterbody *via* a river or a stream	0.12	0.04
4	Connectivity to another EWM invaded waterbody *via* a river or a stream	0.09	0.10
5	Eigenvector centrality of the boater traffic network	0.28	0.34
6	Inverse of the Euclidean distance to the nearest major road	0.21	0.09

*ZM, zebra mussels; EWM, Eurasian watermilfoil*.

**Table 3:** Summary of co-kriging model validations for the probability of zebra mussel (ZM) and Eurasian watermilfoil (EWM) introductions in Minnesota.

		AUC	Sensitivity at risk rank 3	Specificity at risk rank 3
Cross validation	ZMs	0.73	0.70	0.63
	EWM	0.79	0.82	0.74
True validation	ZMs	0.78	0.78	0.72
	EWM	0.76	0.83	0.61

*Cross validation was done using the k fold test (k = 5). True validation was done by fitting models for invasions as of 2010 and validating using the invasions reported between 2011 and 2015. Area under the receiver operating characteristic curve (AUC), sensitivity, and specificity at the threshold risk are summarized*.

Output maps for both ZM and EWM co-kriging and the number of waterbodies classified under each risk rank are seen in Figures 1 and 2. Figure 1 illustrates the risk maps for the models fitted for the invasions by 2010, whereas Figure 2 shows the risk based on the invasions by 2015. Therefore, by 2015, at the risk rank = 5, a total of 2,293 (12.45%) and 2,289 (12.43%) waterbodies were at very high risk of invasion by ZMs and EWM, respectively. Among the waterbodies at very high risk at risk rank 5 for both the AIS, 755 waterbodies were in common. Therefore, a total of 3,827 (20.78%) waterbodies were at high risk for either ZM or EWM invasions.

## Discussion

This study was aimed at predicting the risk of ZMs and EWM invasions in Minnesota using network analysis and co-kriging, a geostatistical modeling technique. Recognizing areas at high risk for invasion may facilitate early detection and efficient control through risk-based management. This study emphasized the use of co-kriging on observed data affected by underreporting and other reporting biases by augmenting the predictive power of one variable with the support of other correlated and highly sampled variables. In the absence of active surveillance, invasions are recorded based on public reporting and subsequent confirmation by the MNDNR. Therefore, presence of the AIS may be missed in some waterbodies due to insufficient coverage, resulting in underreporting. Results suggested that, by 2015, nearly 20% of the waterbodies in Minnesota were at high risk of invasions by either or both AIS. This included 2,293/18,411; 12.45% waterbodies at risk of ZM invasions and 2,289/18,411; 12.43% waterbodies at risk of EWM invasions, whereas only 125/18,411 (0.67%) and 304/18,411 (1.65%) confirmed the invasions, respectively. Recognition of areas at high risk may act as an early warning system and help prioritization of water bodies for risk-based surveillance and management.

The key predictors of the best fitted co-kriging models, for both ZMs and EWM, were the distance to the nearest infested location and the boater traffic, i.e., Eigenvector centrality of the boater traffic network. This result emphasizes the proximity between waterbodies and human-mediated dispersal as useful predictors of potential invasions (7, 9). The strong relationship between hitchhiking ZM larvae along with the residual water, boat equipment, and recreational gear is a known risk factor for invasions (13). Affirmatively, the secondary variables in the final co-kriging model for ZMs were both indicators of human-mediated dispersal of the AIS, the boater traffic and the distance to the nearest major road which represents the convenience for frequent accessibility. The final co-kriging model for EWM suggests that their distribution is attributable to the proximity between waterbodies as determined by the invasion status of EWM, the natural dispersal *via* connecting surface water such as rivers and the human-mediated transportation (i.e., variables 2, 4, and 5). The predictive power of the boater traffic using the Eigenvector centrality measure is augmented with the use of the inverse distance to the nearest major road as a secondary predictor, which adjusted for the potential underreporting. The Pearson correlation between ZM invasions and the inverse of the distance to the nearest major road was 0.21 (Table 2), which was stronger than other variables. Distance to the nearest major road represents the convenience of frequent accessibility to the waterbody.

In the absence of active surveillance, AIS invasions are recorded based on public reporting and subsequent confirmation by the MNDNR (5). Therefore, densely human populated areas are likely to be reported with invasions more frequently than less populated areas, where underreporting is possible (14; see text footnote 1). Considering the commonalities between waterbodies with currently reported invasions and searching for waterbodies with similar characteristics using waterbody-specific variables may be one of the solutions to correct for underreporting (25). However, selecting waterbodies based on multiple criteria such as public water access, boater traffic, and tourist activity. is challenging and through this study we provide a method which take the most correlated variables into account and produce risk maps and risk ranks for each waterbody, which may offer a better guidance to volunteers who search for potential invasions. This approach of risk-based and targeted surveillance would provide more opportunities to reduce the problem of underreporting.

An important strength of the present study is that the boater traffic was calculated at the waterbody level. This is more informative compared to the representation of boater movement by county centroids, such as the studies by Stewart-Koster et al. (22) and Buchan and Padilla (12). Representation of the boater traffic by county leads to either overestimation or underestimation of the importance of individual waterbodies (22).

Areas at high risk for AIS infestations may be identified using a variety of modeling techniques. Species distribution modeling (42), diffusion models (43), gravity models (44), regression models (12), machine learning techniques (45), risk models (46), and model combinations (22) are approaches commonly used for the estimation of AIS distribution risk. Some of the abovementioned computationally complex modeling techniques are powerful when determining the risk of invasions; however, the complexity of these models can make the translation of the model output into practice a difficult task. Compared to above modeling techniques, co-kriging is a less complicated analysis. When translating the science to policy, the concept of using correlated and highly sampled variables to estimate unknown variables is rather simple and straightforward. Therefore, the use of co-kriging as an introductory tool to assess the risk and introducing the method to the decision-makers perhaps is a step further into translating science into practice.

One limitation of our approach is that co-kriging interpolation assumes that the probability of AIS introduction is a continuous variable across geographical space (15). However, the probability of AIS introduction is waterbody specific and not a continuous variable. In this study, the assumption of continuous probability may be justified because Minnesota is a water rich state with over 19% of the state is consisting of lakes, ponds, rivers, and wetlands (27). This assumption of continuous probability is also supported by the density and complexity of the overland boater traffic (Figures S1 and S2 in Supplementary Material). Although this simplification of continuous probability is held commonly in spatial modeling (20), the invasions only occur at the susceptible locations, i.e., the waterbodies. In co-kriging, probability is computed for cells and, here, we assumed the probability of infection to be 0 for those cells in which no waterbody was found, whereas the probability of AIS introduction was computed for cells that was occupied, at least in part, by a waterbody. Presentation of co-kriging models in the format of isopleth maps with a continuous probability surface is common in the spatial modeling (20). As mentioned in the methods, magnitude and the duration of the infestation would have been ideal to be included in the analysis because it is a measure of the risk an infested waterbody pose on susceptible waterbodies (9). However, magnitude of invasions was not readily available because the collection of magnitude of invasions is a costly and labor-intensive process (47, 48) and the distribution of AIS within waterbodies is patchy based on the substrate compositions (48, 49). Similarly, the assignment of surface water connectivity both upstream as well as downstream, without limiting the distances, may lead to potential overestimation of the risk of invasion. However, assignment of distance limits of upstream and downstream transmission was subjective as described by multiple studies (28, 31, 32). Another limitation is the lack of AIS distribution data in the states adjacent to Minnesota, which is important for effective cross-boundary control and preventive measures. For example, waterbodies in east central Minnesota are affected by both ZMs and EWM. However, the study described by Stewart-Koster et al. (22) indicated low risk of the ZM and EWM invasion across the border in northeastern Wisconsin (22). Our study does not account for ZMs and EWM invasions in the adjacent states either, which indicates the risk of invasion may have been underestimated. Being confined within the political boundaries often results in reducing the model accuracies (50). The geographical area for the analysis was not expanded to the Midwest or great lakes because some of the required data, such as boater movement, was not available from all the locations.

As seen in Figure 2, a total of 5,458 (29.64%) of the waterbodies were recognized to be equal or above the threshold risk rank 3 for ZM invasions. Similarly, 7,119 (38.66%) of the waterbodies were predicted to be above the risk rank 3 for EWM invasions. From a management stand point, these numbers of waterbodies are still too high to plan a cost-effective risk-based surveillance or develop targeted management plans. Therefore, risk-based management using limited resources requires prioritizing the waterbodies at high risk for screening (21, 24). This inherent difficulty of recommending sample sizes to be collected from risk regions is also discussed by another study where co-kriging was used to conduct a *post hoc* comparison of the association between highly pathogenic avian influenza (H5N1) incidences and intensity of surveillance activities of sampling wild birds by administrative region (20). Resource availability, degree of risk awareness, and participation in reporting by the region were recognized as key factors defining the extent of surveillance efforts (20). We suggest focusing on the waterbodies of biological and recreational importance. This can be a value-based judgment and should include a variety of stakeholders and agreed upon criteria. Prioritization of the waterbodies could also be done by conducting a risk-based survey by subdividing the counties into smaller polygons or using township areas. One such approach is the hexagonal tiling method, which is commonly used in ecological studies (51). The risk rank generated from this study may also be useful to improve the MNDNR’s Watercraft Inspection Program by recruiting watercraft inspectors at areas recognized to be at high risk for invasions and not currently inspected.

Risk-based management is not a novel concept (21, 26). However, the attempt to incorporate spatial models in invasion risk assessment to inform the decision and policy-making process may improve the efficiency and effectiveness of the AIS control programs, through targeted and risk-based sampling schemes (23, 24). As demonstrated here, co-kriging enables predicting values for locations without complete data, using correlated and highly sampled variables, which can be used as a solution to the underreporting in ecological and epidemiological studies. This work seeks to encourage the use of scientifically supported quantitative procedures such as network analysis and co-kriging to solve the problem of imperfect detections, which subsequently improve the early detection of biological invasions.

## Author Contributions

KK conducted the data mining, data analysis, and the manuscript writing. MA edited the manuscript. NP contributed in obtaining data, interpretation of the results, and manuscript editing. AP consulted the data analysis, troubleshooting of the method, and manuscript editing.

## Conflict of Interest Statement

The authors declare that the research was conducted in the absence of any commercial or financial relationships that could be construed as a potential conflict of interest.

## Footnotes

^1^Kanankege KST, Alkhamis MA, Perez AM, Phelps NBD. Zebra mussels and Eurasian watermilfoil detection patterns in Minnesota (2017). Under review.

## Supplementary Material

Figure S1 The boater traffic between waterbodies based on the Watercraft Inspection Program conducted by Minnesota Department of Natural Resources. The data from year 2013 are illustrated. Panel **(A)** represents the movement of boaters from previously visited waterbody-to-waterbody where the survey data were collected. Panel **(B)** represents the movement of boaters from waterbody where the survey data were collected-to-the waterbody where they plan to visit next.

Figure S2 An illustration of the Eigenvector centrality for the waterbodies in the boater traffic network created using the surveys of Watercraft Inspection Program conducted by Minnesota Department of Natural Resources. The data from year 2013 are illustrated.
