# Supplementary figures and images for: A Probability Co-Kriging Model to Account for Reporting Bias and Recognize Areas at High Risk for Zebra Mussels and Eurasian Watermilfoil Invasions in Minnesota
Source: Front Vet Sci. 2018 Jan 4;4:231. doi: 10.3389/fvets.2017.00231 (PMC5758494; doi:10.3389/fvets.2017.00231)

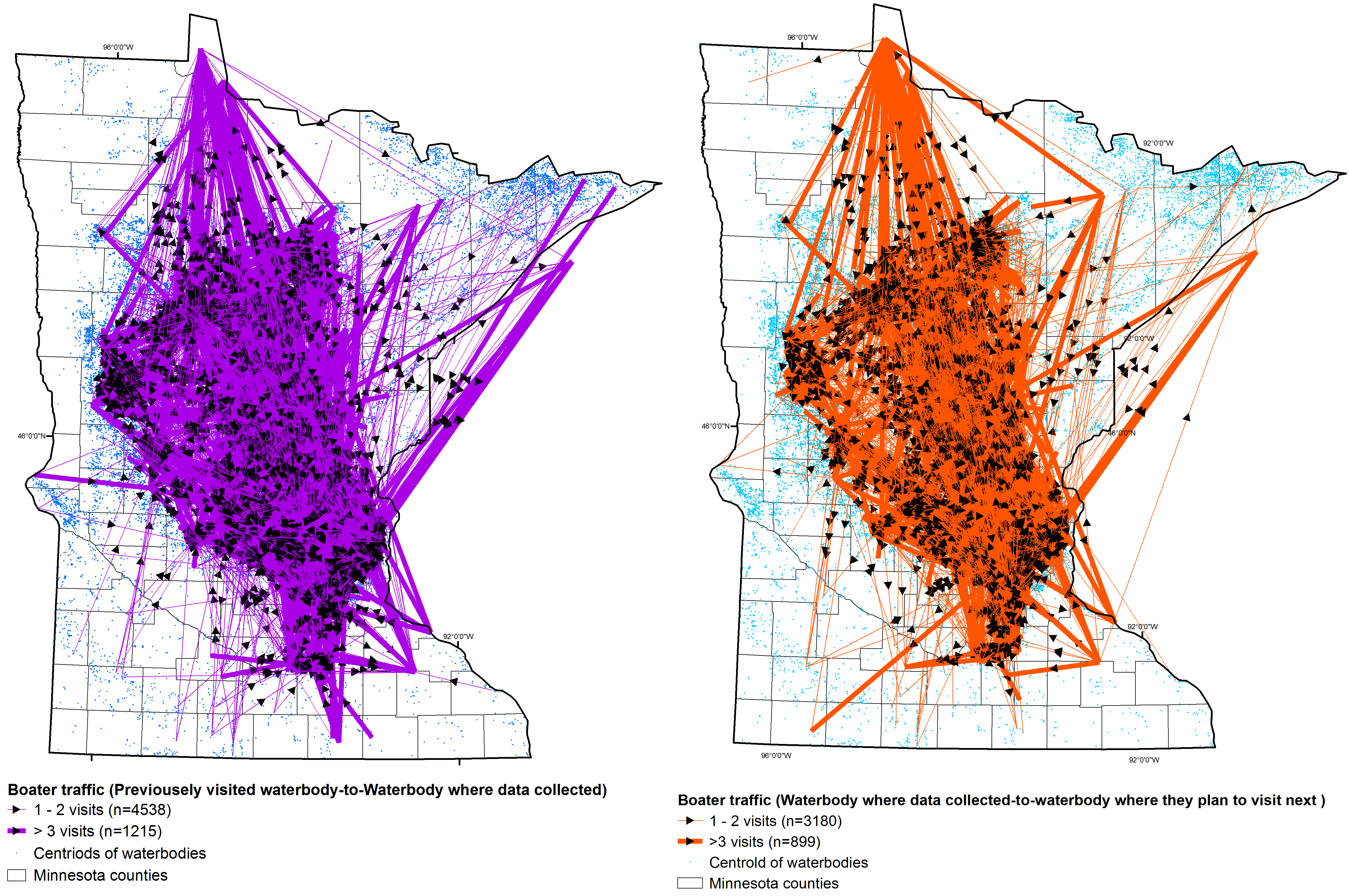

Supplement: Figure S1 — The boater traffic between waterbodies based on the Watercraft Inspection Program conducted by Minnesota Department of Natural Resources. The data from year 2013 are illustrated. Panel (A) represents the movement of boaters from previously visited waterbody-to-waterbody where the survey data were collected. Panel (B) represents the movement of boaters from waterbody where the survey data were collected-to-the waterbody where they plan to visit next. [file Image_1.tif]

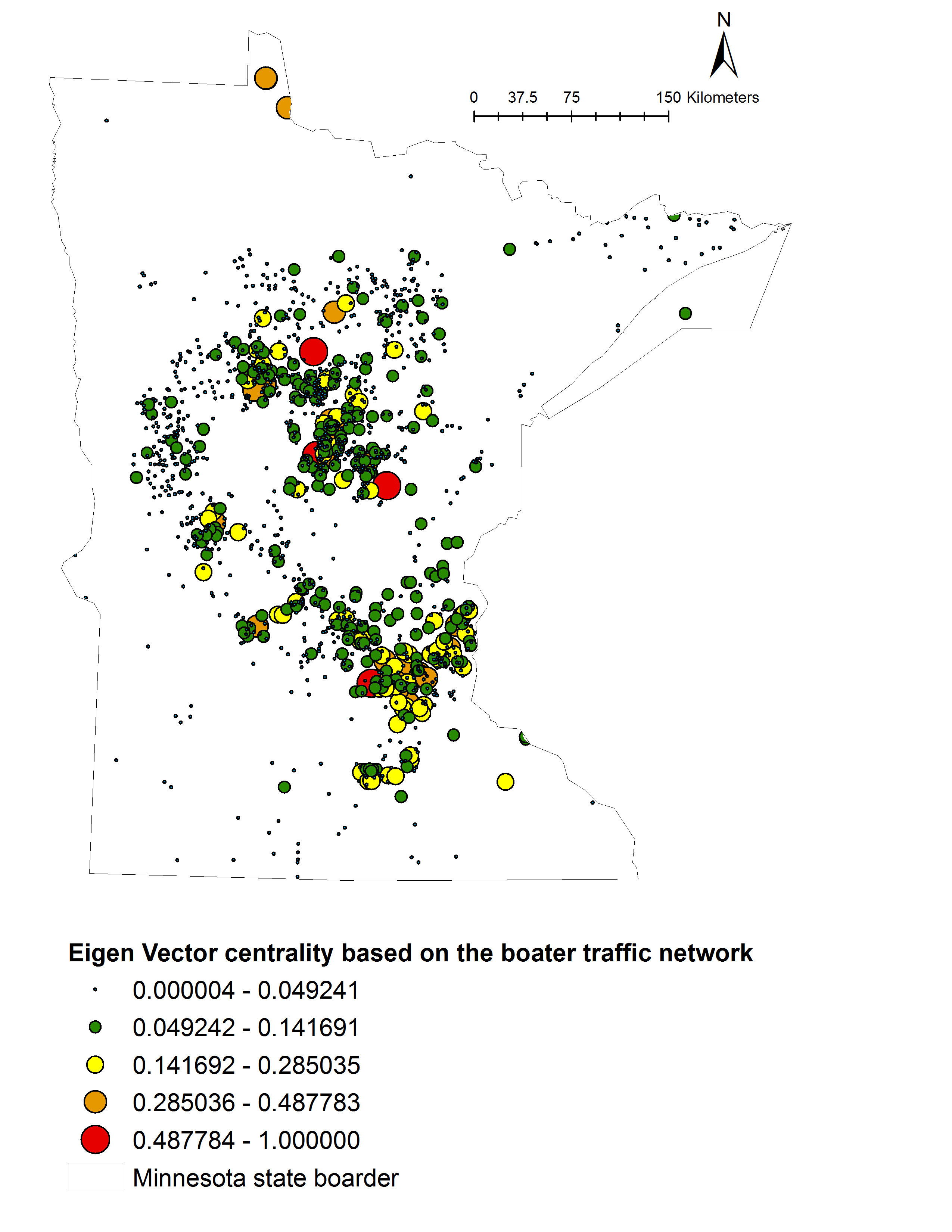

Supplement: Figure S2 — An illustration of the Eigenvector centrality for the waterbodies in the boater traffic network created using the surveys of Watercraft Inspection Program conducted by Minnesota Department of Natural Resources. The data from year 2013 are illustrated. [file Image_2.tif]
