# Supplementary material for: A Probability Co-Kriging Model to Account for Reporting Bias and Recognize Areas at High Risk for Zebra Mussels and Eurasian Watermilfoil Invasions in Minnesota
Source: Front Vet Sci. 2018 Jan 4;4:231. doi: 10.3389/fvets.2017.00231 (PMC5758494; doi:10.3389/fvets.2017.00231)
Supplement: Supplementary file 3 [file Table_1.docx]

**Supporting Information**

**Table S1**. Parameters used in the co-kriging models for Eurasian watermilfoil (EWM) and zebra mussels (ZM).

| Parameter | ZM model^*^ | | | EWM^#^ model | | |
| --- | --- | --- | --- | --- | --- | --- |
| Lag size | 0.025 | | | 0.04 | | |
| Number of lags | 12 | | | 8 | | |
| Models | Exponential | | | Exponential | | |
| Major range | 0.15 | | | 0.15 | | |
|  |  | | |  | | |
| Search neighborhood parameters for each predictor variable | Primary variable: ZM invasion status | Boater traffic | Inverse of the Euclidean distance to nearest major road | Primary variable: EWM invasion status | Boater traffic | Connectivity to another EWM invaded waterbody via a river or a stream |
| Minimum number of neighbors to be included in the kriging process | 5 | 200 | 200 | 50 | 50 | 200 |
| Maximum number of neighbors to be included in the kriging process | 2 | 2 | 2 | 2 | 2 | 2 |
| Sector type | 1 | 1 | 1 | 1 | 1 | 1 |
| Angle | 135 | 135 | 135 | 120 | 120 | 120 |
| Anisotropy factor | 2 | 1 | 1 | 1 | 1 | 1 |
| Root mean Square value (RMS) of the model | 0.057 | | | 0.1058 | | |
| RMSE standardized value of the model | 3.86 | | | 0.836 | | |
| Nugget | 0 | | | Between 0 and 0.01 | | |
| Major range | 0.15 | | | 0.15 | | |
